# Prevalence of Dental Fluorosis among Southern Jordanian Population

**DOI:** 10.1155/2020/8890004

**Published:** 2020-10-29

**Authors:** Amjad M. Al Warawreh, Zaid H. Al Tamimi, Mohammad I. Al Qatawna, Aseel A. Al Momani, Mohammed R. Al Mhaidat, Waddah S. El Naji, Salem AlSaraireh

**Affiliations:** Royal Medical Services, Amman, Jordan

## Abstract

**Introduction:**

Jordan is one of the Middle Eastern countries that is classified as a poor water resource country. People in Jordan consume any available water. In the south of Jordan, water resources are limited. The drinking water contains high levels of fluoride, which in turn leads to augmented danger of both skeletal and dental fluorosis.

**Aims:**

This study is focused on evaluating the pervasiveness of dental fluorosis among patients of Karak City and assessing the degree and distribution of fluorosis.

**Materials and Methods:**

This research focuses on 2,512 patients ranging from 12 to 52 years old seeking dental treatment in the Dental Department at “Prince Ali ben Al Houssin Hospital” in Karak City. Dental fluorosis status was assessed by using Modified Dean's Fluorosis Index.” The data collected were subjected to statistical analysis.

**Results:**

The dental fluorosis prevalence within our sample was 39.9% in Karak City. Females were more influenced than males, and fluorosis was detected more often in those who drank tap water and was more common in a very mild and localized form.

**Conclusion:**

Fluorosis necessitates constant observation, and future study in terms of the intake in Jordan is recommended in terms of all sources. It would not be too soon to note that the supply of drinking water needs to be changed in South Jordan.

## 1. Introduction

Fluorine is a natural element that has been observed to exist in many mineral forms. Geographic activity such as weather and volcanoes may cause increased its quantity within drinkable water [1]. In the human body, fluorine plays a vital part when it comes to the mineralization of teeth, bones, and other hard tissues. The normal mineralization of bone and dental formation requires small quantities of fluorine which can be obtained from drinking water and consuming food such as seafood, cheese, and tea. Prolonged or excess exposure to high concentrations of fluorine [2, 3] can be toxic, and high concentration of fluoride in serum can impair the skeletal system by calcification of muscular attachments, ligaments, and ossification on histopathologically [4]. Some excess concentration of fluoride can lead to a sluggish and progressive health issue called fluorosis [5], and milk intake shows reduction in severity of symptoms [6]. In many countries in the Middle East, it is seen as a significant health issue [7].

There are few reports on the effect of fluorosis on periodontal health in the literature, but there are some epidemiological studies concerning the prevalence of periodontal disease among population with dental fluorosis, and they show high level of inflammation in fluorosis than nonfluorosis areas [8–11]. On the other hand, some other studies show no relation [12, 13] and others studies show better periodontal status in high fluoridated area [12, 14, 15]. Latest studies show that the presence of fluoride ion has an effect on bacterial and microorganism reduction which affects periodontal status indirectly by reducing the inflammatory process similarly to some other factors such as nutraceutical agents, transforming factor-*β* (TGF-*β*), vascular endothelial growth factor (VEGF), and asymmetric dimethylarginine (ADMA) [16–19]. An appropriate level of fluoride ion within salivary fluid causes enamel demineralization reduction [20–22].

The World Health Organization (WHO) holds that the highest allowed level of the substance in water should not exceed 1.5 mg/l so that issues with bones and teeth can be avoided. “Dental fluorosis” is a condition where both tooth aesthetics and formation are impacted because of a chronic presence of fluoride. Both the enamel development and mineralization are disrupted at the level of intracellular and extracellular [23, 24], and the presence of lesion stemming from fluorosis itself is link to substantial consumption of the same within the critical phase (postsecretory or early maturation phase) where the development of the tooth itself is taking place. At the microscopic level, fluorosis affects the enamel formation by making it more porous. As a result, the greater the fluoride content in the enamel, the more porous the enamel becomes [23, 25–28]. At the structural level, the enamel crystals are arranged normally, but an increase in intercrystalline space causes an increase in porosity [23, 29, 30]. Many epidemiological studies consider that these symptoms are important risk factors associated with other systemic diseases [25, 29, 31–45].

Dental fluorosis had differences in the susceptibility and severity among population. This could be due to genetic variation. Matrix metalloproteinase 20 (MMP20) gene variation was present in population with high exposure to fluoride in drinking water and associated with the less sever phenotypes of dental fluorosis. Thus, single-nucleotide variations (SNVs) considered a marker for lesser dental fluorosis susceptibility [46].

The tooth surface and distribution of fluorosis in the mouth have a very characteristic appearance [23, 25, 39, 40, 47–49]. Fluorosis can develop from birth to eight years old, and the aesthetics of teeth can be affected from birth to six years old. Premolars typically present a larger occurrence of the issue and face a significant amount of damage [33, 35, 50]. In clinical terms, enamel fluorosis presents as white opaque lines or spots and can even show up such as a white parchment on the surface of the tooth itself. At times, severe-to-moderate fluorosis has been observed, and brown stains may also show up because of the absorbing of extrinsic stains caused by the food intake. Discrete pitting is also seen in severe fluorosis at higher fluoride concentrations accompanying extrinsic stains. The distribution of the fluorosis is symmetrical, but severity varies [25, 31–33, 35–38]. As treatment option, the mild form rarely needs treatment, especially if the posterior teeth were the affected one, but for moderate-to-severe cases, especially the teeth were within aesthetic zone. Treatment ranges from microabrasion technique and bleaching or could be extended to resin covering or even partial and complete coverage (veneer and full crown) [35, 51, 52]. Regarding prevalence of dental caries among patients with fluorosis, still there is no clear evidence of any significance relation but more related to oral hygiene level [32]. In 2003, Hamdan et al. investigated dental fluorosis in multiple cities and found a high prevalence in South Jordan. More investigation is required in these areas [53]. The study deals with how prevalent the issue is in South Jordan, where drinking water is unmonitored for fluoride concentration. The region is geographically close to a phosphate factory which increases fluoride concentrations in the surrounding environment due to the phosphate-manufacturing process.

The aim of this study is to observe the prevalence of fluorosis among population of Karak City and compare it with previous study in the same region and worldwide to confirm or reject the pattern that occurs within last decades with increased fluoride uptake due to food, tooth paste, or industrial evolution and uses synthetic fertilizers in agriculture.

## 2. Materials and Methods

This study looked at 2,512 patients aged between 12 and 52 years (1,158 males and 1,354 females) who sought dental treatment in the Dental Department at Prince Ali ben Al-Hussein Hospital in Karak City between March and December 2018. The Ethics Committee of the Royal Medical Services approved the study. Verbal consent was obtained from each patient by the operator.

Data were recorded from examinations performed by four dentists over a period of 10 months. The training and examination procedures were standardized and calibrated between the examiners by using the same index and by re-examining a sample of patients by different dentists; in addition, one month later, we re-examined the same patients by same dentists who did the previous examination to ensure reliability. Patients provided demographic information through a questionnaire that included personal data such as name, age, gender, social number, and the source of drinking water. All the recordings were obtained in natural daylight using a mouth mirror and following standard infection control guidelines [54]. The Modified Dean's Fluorosis Index was used for dental assessment [55]:

- Unaffected (normal): the enamel had a translucent appearance, and the tooth surface exhibits a glossy, smooth appearance. The colour of such a tooth holds a pale or white shade.
- Questionable: here, the enamel presents some changes from the discussion above. The tooth can present an occasional white fleck or spots. This applies in cases where “definitive determination of the mildest form of fluorosis is not warranted and a classification of unaffected is not justified.”
- Very mild: “small opaque paper-white areas are scattered over the tooth surface but do not involve as much as 25% of the surface.”
- Mild: “white opaque areas on the surface are more extensive but do not involve as much as 50% of the surface.”
- Moderate: 50% of the surface presents white opaque patches.
- Severe: the entirety of the tooth's enamel is impacted. This classification is marked by confluent or discrete pitting.

Statistical Package of Social Science (version 17, SPSS Inc., Chicago, IL, USA) was used to develop the statistical analyses. The study also deployed descriptive statistics to expand on severity, prevalence, and distribution of dental fluorosis.

## 3. Results

**Figure 1:**
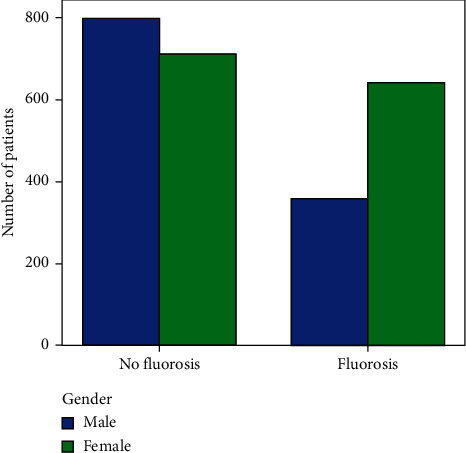
Incidence of fluorosis grouped by gender.

**Figure 2:**
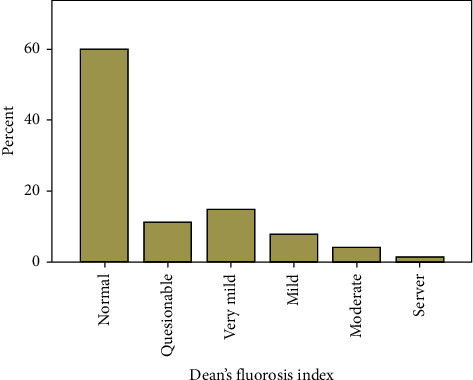
Distribution of fluorosis according to the Dean's Fluorosis Index.

**Figure 3:**
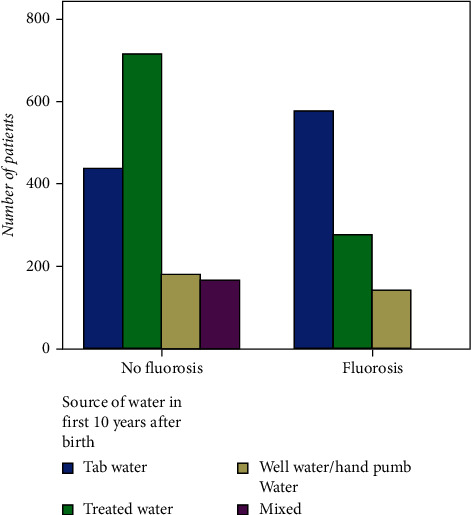
Incidence of fluorosis grouped by source of water in first 10 years after birth.

The study sample was made up of 2,512 patients (46.1% male; 53.9% female) with a mean age of 21.5 ± 9.7 years. A total of 1,002 patients (39.9%) had some extent of fluorosis, and it was more commonly observed in females when looking at the majority in the 12- to 30-year-old group (Table 1 and Figure 1). Localized fluorosis was more common (44.7% of the total sample; 67.4% of patients with fluorosis) (Table 2). The fluorosis index ranged dramatically from the lowest and least severe (very mild: 15.0% of the total sample and 37.6% of patients with fluorosis) to the highest index (severe form: 1.6% of the total sample and 3.9% of patients with fluorosis) (Table 3 and Figure 2). Fluorosis was more common in patients who drank tap water (56.8%) and least common in patients who drank treated water (28.1%) in their first 10 years of their life (Table 4 and Figure 3).

## 4. Discussion

This study was focused on gauging how prevalent the issue was within Karak City, a southern area of Jordan with prevalent fluorosis, and to measure the fluorosis distribution according to gender, drinkable source of water, and its extent and influence. Unsurprisingly, fluorosis caused a rise in the severity and prevalence as its content in drinkable water went up. Moreover, it had a considerably high prevalent in optimal areas. On the other hand, the likelihood of it occurring in this study presented a clear similarity to studies in the United States of America (USA) and Mexico which show increase in prevalence of fluorosis [56, 57]. Hamdan et al. had performed a study in Jordan several years ago. [53]. Furthermore, no major improvements have been undertaken to improve the water source in this area [53]. Interestingly, in 1989, Fraysse et al [58] found that the number stood at a significant 80%. The result is much higher than that reported within the research at hand. There was an apparent variation of the fluorosis prevalence discussed under this study in the context of data from other Arab nations (Table 5).

Under a study conducted by Rugg-Gunn et al. [59] on 14-year-olds in Riyadh, the result showed an 83% enamel mottling among the participants. Akpata et al. [60] studied Hail in Saudi Arabia and observed a result of 90% among children of school-going age. Vigild et al. [61] looked at low fluoride areas in Kuwait and found a 6% prevalence when looking at the 12–15 age group. The result is significantly smaller than that of this research.

Sudan has an endemic when it comes to dental fluorosis. This is true even for areas with low fluoride. Ibrahim et al. [62] conducted a study that showed results between 91 and 100%, where 91% was noted in low areas and 100% in high ones. The difference in prevalence is a partial reflection of the difference in how diagnostic criteria are applied, the method of sampling, or the quantity of fluoride consumed from multiple places.

Fluorosis seems to have a higher trend today than in the period between the 1940s and 2010s [56, 63]. The rise can be attributed to the increase in the number of sources with the substance, including topical applications, dietary supplements, dental hygiene rinses, and dentifrices. However, no substantial body of evidence proves that these elements impact or influence high prevalence of mottling within this work; this is especially true for South Jordan (Karak, Tafila, and Ma'an); furthermore, the sources mentioned are not accessible for children.

Temperature variation can have an impact on severity when it comes to the South. It has been observed that when the highest daily temperature experiences a hike, the water intake does as well [64]. Drinking water, therefore, could also see an increase for children when temperatures hit a mean of 23°C. Consuming water is the easiest solution to such temperatures because it is inexperience and readily available, unlike other sources. This aspect could have a role in the heightened level of the substances being consumed by children.

Phosphate mines are another significant contributor to the issue. Around 60% of the land in the country is covered in these mines, given that it is the fifth largest producer of phosphate rock. Jordan is also the second largest exporter in the world when it comes to these rocks. These rocks can contain between two and four percent fluorine [65]. This is seen as a significant element impacting the severity and high prevalence of fluorosis. In Tafila, Ma'an, and Karak, fluorides can be emitted in solid and gaseous forms. When in solid state, they are emitted as particles, whereas the gas is produced as silicone tetrafluoride and hydrogen fluoride. Plants that are covered in these particles or have absorbed this as a gas can eventually affect the human respiratory system.

Team is also a high contributor to the issue. Many people that live in South Jordan fall within lower-income groups and are classified as poor. Children consume a considerable amount of tea, and it is the liquid they consume the most after water. It has an impact to the total amount of fluoride that children will consume. Fraysse [58] was of the view that the 80% result of their study found was linked to the high annual mean temperature, which caused the subjects to drink more water and therefore augment their intake of fluoride as a result. Estimates show that tea can add around 2.7 mg of fluoride on a daily basis to a child's diet, alongside that of an adult [66, 67].

Females were shown to be more susceptible to fluorosis than males, which corresponds with previous studies. In addition, fluorosis is most common in the 12-20-year-old patients (89.3% of total patients with fluorosis; 59.0% of the group) [53].

Fluorosis can alter tooth surface and cause pitting and porosity which enhance the adherence of bacteria which in role increase inflammatory process and cause gingival inflammation; in addition to effect on cementocytes and the formation of hypercementosis in roots which may cause difficulty in scaling and root planning, this may affect periodontium directly or indirectly [68, 69]. Having optimal fluoride level can positively affect the periodontal health by reducing the bacterial growth and gingival inflammation [70].

According to severity and location, dental fluorosis cosmetic awareness of patients can be well noticed and needs to be dealt with. A lot of studies regarding the best of treatment still had some controversy ranging from conservative procedures (such as microabrasion and bleaching) to nonconservative one (such as veneer and full crown) finally with in between (Resin coverage) or even no treatment in mild case or when affected tooth/teeth away from aesthetic zone with advantage and disadvantage for each procedure [35, 52, 71].

In conclusion, the prevalence of fluorosis requires continuous monitoring and further investigation is needed into the sources that contribute to total fluoride intake in Jordan. Whilst drinking water is the major factor contributing to fluoride intake, we must consider the contribution from multiple sources such as tooth paste and any industrial waste and pollution with fluorine element. In addition, health education and community awareness for preventing fluorosis are needed, for early intervention to reduce the consequences on dental and periodontal health status.

## Figures and Tables

**Table 1:** Incidence of fluorosis grouped by patient age.

Age group (years)	Fluorosis		
	No fluorosis	Fluorosis	Total

12–20	622	896	1518
21–30	449	95	544
31–40	295	11	306
>40	144	0	144
Total	1510 (60.1%)	1002 (39.9%)	2512

**Table 2:** Location of incidence of fluorosis.

	Location of fluorosis			
	Localized	Generalized	Normal	Total
No fluorosis	0	0	1510	1510
Fluorosis	675	327	0	1002
Total	675	327	1510	2512

**Table 3:** Distribution of fluorosis according to the Dean's Fluorosis Index.

	Fluorosis index						
	Normal	Questionable	Very mild	Mild	Moderate	Severe	Total
Percentage	1510	280	377	202	104	39	2512
	60.1 %	11.1 %	15.0 %	8.0 %	4.1 %	1.6 %	100 %

**Table 4:** Incidence of fluorosis grouped by source of water in first 10 years after birth.

	Source of water in first 10 years after birth				
	Tab water	Treated water	Well water/hand plumb water	Mixed	Total
No fluorosis	440 (43.2%)	717 (71.9%)	183 (56.1%)	170 (100.0%)	1510 (60.1%)
Fluorosis	579 (56.8%)	280 (28.1%)	143 (43.9%)	0 (0.0%)	1002 (39.9%)
Total	1019 (100.0%)	997 (100.0%)	326 (100.0%)	170 (100.0%)	2512 (100.0%)

**Table 5:** Incidence of fluorosis in the region.

	Al Warawreh et al. Jordan (Karak) 2020 (%)	Hamdan et al. Jordan (Karak) 2003 (%)	Fraysse et al. Jordan 1989 (%)	Rugg-Gunn et al. Saudi Arabia Riyadh (%)	Akpata et al. Saudi Arabia Hail (%)	Vigild et al. Kuwait (%)	Ibrahim et al. Sudan (%)
Percentage of fluorosis	39.9	39.0	80.0	83.0	90.0	6.0	91.0

## Data Availability

The data used to support the findings of this study are currently available upon request, and 12 months after publication of this article, the request will be considered by the corresponding author.

## References

[1] Akuno M. H., Nocella G., Milia E. P., Gutierrez L. (2019). Factors influencing the relationship between fluoride in drinking water and dental fluorosis: a ten-year systematic review and meta-analysis. *Journal of Water and Health*.

[2] Khichar M., Kumbhat S. (2015). Defluoridation-a review of water from aluminium and alumina based compound. *International Journal of Chemical Studies*.

[3] Zuo H., Chen L., Kong M. (2018). Toxic effects of fluoride on organisms. *Life Sciences*.

[4] Reddy D. B., Mallikharjunarao C., Sarada D. (1969). Endemic fluorosis. *Journal of the Indian Medical Association*.

[5] Kumar A., Kumar V. (2015). Fluoride contamination in drinking water and its impact on human health of Kishanganj, Bihar, India. *Research Journal of Chemical*.

[6] Rango T., Kravchenko J., Atlaw B. (2012). Groundwater quality and its health impact: an assessment of dental fluorosis in rural inhabitants of the main Ethiopian rift. *Environment International*.

[7] Arlappa N., Aatif Qureshi I., Srinivas R. (2013). Fluorosis in India: an overview. *International Journal of Research & Development of Health*.

[8] Vora K., Vora P. (2013). Assessment of periodontal status of the patients with dental fluorosis in area with natural high levels of fluoride: a cross-sectional survey. *Dental Hypotheses*.

[9] Waweru L. W., Kimani H., Opinya G. N., Ng’ang’a P. M. (2015). Periodontal status of children with dental fluorosis in Juja, Kenya. *American Journal of Medicine and Medical Sciences*.

[10] Massler M., Schour I. (1951). Relation of malnutrition, endemic dental fluorosis and oral hygiene to the prevalence and severity of gingivitis. *Journal of Periodontology*.

[11] Day C. (1940). Chronic endemic fluorosis in northern India. *British Dental Journal*.

[12] Haikel Y., Turlot J.-C., Cahen P.-M., Frank R. (1989). Periodontal treatment needs in populations of high- and low-fluoride areas of Morocco. *Journal of Clinical Periodontology*.

[13] Zimmermann E. R., Leone N. C., Arnold F. A. (1955). Oral aspects of excessive fluorides in a water supply. *The Journal of the American Dental Association*.

[14] Englander H. R., Kesel R. G., Gupta O. P. (1963). The Aurora-Rockford, Ill., study II. effect of natural fluoride on the periodontal health of adults. *American Journal of Public Health and the Nations Health*.

[15] Russell A. L. (1957). Fluoride domestic water and periodontal disease. *American Journal of Public Health and the Nations Health*.

[16] Fiorillo L., Cervino G., Herford A. S., Laino L., Cicciù M. (2020). Stannous fluoride effects on enamel: a systematic review. *Biomimetics*.

[17] Isola G., Polizzi A., Iorio-Siciliano V., Alibrandi A., Ramaglia L., Leonardi R. (2020). Effectiveness of a nutraceutical agent in the non-surgical periodontal therapy: a randomized, controlled clinical trial. *Clinical Oral Investigations*.

[18] Matarese G., Isola G., Anastasi G. P. (2012). Immunohistochemical analysis of TGF-*β*1 and VEGF in gingival and periodontal tissues: a role of these biomarkers in the pathogenesis of scleroderma and periodontal disease. *International Journal of Molecular Medicine*.

[19] Isola G., Alibrandi A., Currò M. (2020). Evaluation of salivary and serum asymmetric dimethylarginine (ADMA) levels in patients with periodontal and cardiovascular disease as subclinical marker of cardiovascular risk. *Journal of Periodontology*.

[20] Kanduti D., Sterbenk P., Artnik a. (2016). Fluoride: a review of use and effects on health. *Materia Socio Medica*.

[21] Buzalaf M. A. R., Pessan J. P., Honório H. M., Ten Cate J. M. (2011). Mechanisms of action of fluoride for caries control. *Monographs in Oral Science*.

[22] Fincham A. G., Moradian-Oldak J., Simmer J. P. (1999). The structural biology of the developing dental enamel matrix. *Journal of Structural Biology*.

[23] Fejerskov O., Manji F., Baelum V. (1990). The nature and mechanisms of dental fluorosis in man. *Journal of Dental Research*.

[24] Aoba T., Fejerskov O. (2002). Dental fluorosis: chemistry and biology. *Critical Reviews in Oral Biology & Medicine*.

[25] Thylstrup A., Fejerskov O. (1978). Clinical appearance of dental fluorosis in permanent teeth in relation to histologic changes. *Community Dentistry and Oral Epidemiology*.

[26] Küchler E. C., Dea Bruzamolin C., Ayumi Omori M. (2018). Polymorphisms in nonamelogenin enamel matrix genes are associated with dental fluorosis. *Caries Research*.

[27] Bhagavatula P., Levy S. M., Broffitt B., Weber-Gasparoni K., Warren J. J. (2016). Timing of fluoride intake and dental fluorosis on late-erupting permanent teeth. *Community Dentistry and Oral Epidemiology*.

[28] Lins R. B. E., Andrade A. K. M. D., Duarte R. M., Meireles S. S. (2019). Influence of three treatment protocols for dental fluorosis in the enamel surface: an in vitro study. *Rio de Janeiro Dental Journal (Revista Científica do CRO-RJ)*.

[29] Pendrys D. G., Katz R. V. (1989). Risk of enamel fluorosis associated with fluoride supplementation, infant formula, and fluoride dentifrice use. *American Journal of Epidemiology*.

[30] Richards A., Fejerskov O., Baelum V. (1989). Enamel fluoride in relation to severity of human dental fluorosis. *Advances in Dental Research*.

[31] Richards A., Kragstrup J., Josephsen K., Fejerskov O. (1986). Dental fluorosis developed in post-secretory enamel. *Journal of Dental Research*.

[32] Plaka K., Ravindra K., Mor S., Gauba K. (2017). Risk factors and prevalence of dental fluorosis and dental caries in school children of North India. *Environmental Monitoring and Assessment*.

[33] Vivek R., Chaturvedi T. P., Bhatnagar A., Prakash R. (2018). Prevalence OF oral disease in varanasi district-dental caries and dental fluorosis (original study). *Indian Journal of Scientific Research*.

[34] Kumar N., Gauba K., Goyal A., Kapur A. (2018). Comparative evaluation of three different recording criteria of dental fluorosis in a known endemic fluoride area of Haryana. *Indian Journal of Medical Research*.

[35] Di Giovanni T., Eliades T., Papageorgiou S. N. (2018). Interventions for dental fluorosis: a systematic review. *Journal of Esthetic and Restorative Dentistry*.

[36] Do L. G., Ha D. H., Spencer A. J. (2016). Natural history and long‐term impact of dental fluorosis: a prospective cohort study. *Medical Journal of Australia*.

[37] Singh S., Saha S., Singh S., Shukla N., Reddy V. (2018). Oral health-related quality of life among 12-15-year children suffering from dental fluorosis residing at endemic fluoride belt of Uttar Pradesh, India. *Journal of Indian Association of Public Health Dentistry*.

[38] Szpunar S. M., Burt B. A. (1988). Dental caries, fluorosis, and fluoride exposure in Michigan schoolchildren. *Journal of Dental Research*.

[39] Dean H. T., Arnold F. A., Elvove E. (1942). Domestic water and dental caries. V. additional studies of the relation of fluoride domestic waters to dental caries in 4425 white children, aged 12 to 14 years, of 13 cities in 4 states. *Public Health*.

[40] Dean H. T. (1934). Classification of mottled enamel diagnosis. *The Journal of the American Dental Association (1922)*.

[41] Manji F., Baelum V., Fejerskov O. (1986). Dental fluorosis in an area of Kenya with 2 ppm fluoride in the drinking water. *Journal of Dental Research*.

[42] Richards A., Fejerskov O., Baelum V., Likimani S. (1989). Enamel fluoride in unerupted fluorotic human teeth. *Caries Research*.

[43] Larsen M. J., Richards A., Fejerskov O. (1985). Development of dental fluorosis according to age at start of fluoride administration. *Caries Research*.

[44] Evans R. W. (1989). Changes in dental fluorosis following an adjustment to the fluoride concentration of Hong Kong’s water supplies. *Advances in Dental Research*.

[45] Fejerskov O., Yanagisawa T., Tohda H., Larsen M. J., Josephsen K., Mosha H. J. (1991). Posteruptive changes in human dental fluorosis–a histological and ultrastructural study. *Proceedings of the Finnish Dental Society*.

[46] Tremillo-Maldonado O., Molina-Frechero N., González-González R. (2020). DNA sequencing reveals AMELX, ODAM and MMP20 variations in dental fluorosis. *Archives of Oral Biology*.

[47] Dean H. T., Elvove E. (1936). Some epidemiological aspects of chronic endemic dental fluorosis. *American Journal of Public Health and the Nations Health*.

[48] Fejerskov O., Kragstrup J., Richards A. (1988). Fluorosis of teeth and bones. *Fluoride in Dentistry*.

[49] Fejerskov O., Manji F., Bælum V., Møller I. J. (1988). *Dental Fluorosis: A Handbook for Health Workers*.

[50] Baelum V., Fejerskov O, Manji F, Larsen M. J (1987). Daily dose of fluoride and dental fluorosis. *Danish Dental Journal*.

[51] DenBesten P., Li W. (2011). Chronic fluoride toxicity: dental fluorosis. *Monographs in Oral Science*.

[52] Alvarez J. A., Rezende K., Marocho S. M. S., Alves F. B. T., Celiberti P., Ciamponi A. L. (2009). Dental fluorosis: exposure, prevention and management. *Journal of Clinical and Experimental Dentistry*.

[53] Hamdan M. A. M. (2003). The prevalence and severity of dental fluorosis among 12-year-old schoolchildren in Jordan. *International Journal of Paediatric Dentistry*.

[54] World Health Organization (1997). *World Health Organization Oral Health Surveys–Basic Methods*.

[55] Dean H. T. (1942). The investigation of physiological effects by the epidemiological method. *American Association for the Advancement of Science*.

[56] Neurath C., Limeback H., Osmunson B., Connett M., Kanter V., Wells C. R. (2019). Dental fluorosis trends in US oral health surveys: 1986 to 2012. *JDR Clinical & Translational Research*.

[57] Aguilar-Díaz F. D. C., Morales-Corona F., Cintra-Viveiro A. C., De la Fuente-Hernández J. (2017). Prevalence of dental fluorosis in Mexico 2005-2015: a literature review. *Salud Pública de México*.

[58] Fraysse C., Bilbeissi M. W., Mitre D., Kerebel B. (1989). The role of tea consumption in dental fluorosis in Jordan. *Bull Group Int Rech Sci Stomatol Odontol*.

[59] Rugg-Gunn A. J., Al-Mohammadi S. M., Butler T. J. (1997). Effects of fluoride level in drinking water, nutritional status, and socio- economic status on the prevalence of developmental defects of dental enamel in permanent teeth in Saudi 14-year-old boys. *Caries Research*.

[60] Akpata E. S., Fakiha Z., Khan N. (1997). Dental fluorosis in 12-15-year-old rural children exposed to fluorides from well drinking water in the Hail region of Saudi Arabia. *Community Dentistry and Oral Epidemiology*.

[61] Vigild M., Skougaard M., Hadi R. A., Al-Zaabi F., Al-Yasseen I. (1996). Dental caries and dental fluorosis among 4-, 6-, 12- and 15-year-old children in kindergartens and public schools in Kuwait. *Community Dental Health*.

[62] Ibrahim Y. E., Abuaffan A. H., Bjorvatn K. (1995). Prevalence of dental fluorosis in Sudanese children from two villages with 0.25 and 2.56 ppm fluoride in the drinking water. *International Journal of Paediatric Dentistry*.

[63] Clark D. C. (1994). Trends in prevalence of dental fluorosis in North America. *Community Dentistry and Oral Epidemiology*.

[64] Galagan D. J., Vermillion J. R. (1957). Determining optimum fluoride concentrations. *Public Health Reports (1896-1970)*.

[65] Møller I. J., Poulsen S. (1975). A study of dental mottling in children in Khouribga, Morocco. *Archives of Oral Biology*.

[66] Kavanagh D., Renehan J. (1998). Fluoride in tea--its dental significance: a review. *Journal of the Irish Dental Association*.

[67] Smid J. R., Kruger B. J. (1985). The fluoride content of some teas available in Australia. *Australian Dental Journal*.

[68] Murray J. J. (1972). Gingivitis and gingival recession in adults from high-fluoride and low-fluoride areas. *Archives of Oral Biology*.

[69] Vazirani S. J., Singh A. (1968). Endemic fluorosis. Radiological features of dental fluorosis. *Journal of the Indian Dental Association*.

[70] Łukomska-Szymańska M., Zarzycka B., Grzegorczyk J. (2016). Antibacterial properties of calcium fluoride-based composite materials: in vitro study. *BioMed Research International*.

[71] El Mourad A. M. (2018). Aesthetic rehabilitation of a severe dental fluorosis case with ceramic veneers: a step-by-step guide. *Case Reports in Dentistry*.

